# Epidemiology of Sleep Disturbances and Their Effect on Psychological Distress During the COVID-19 Outbreak: A Large National Study in China

**DOI:** 10.3389/fpsyg.2021.615867

**Published:** 2021-05-28

**Authors:** Xilong Cui, Yuqiong He, Jingbo Gong, Xuerong Luo, Jianbo Liu

**Affiliations:** ^1^National Clinical Research Center for Mental Disorders, and Department of Psychaitry, The Second Xiangya Hospital of Central South University, Changsha, Hunan, China; ^2^Department of Applied Psychology, Hunan University of Chinese Medicine, Changsha, China; ^3^Department of Child Psychiatry of Shenzhen Kangning Hospital, Shenzhen Mental Health Center, School of Mental Health, Shenzhen University, Shenzhen, China

**Keywords:** COVID-19, sleep disturbances, gender difference, epidemiology, depression/anxiety/stress

## Abstract

**Background:** The purpose of the current study was to assess the prevalence of sleep disturbances among Chinese people during the COVID-19 pandemic in a large national survey, analyze the relationship between sleep disturbances and mental health status, and explore the influencing factors of the relationship between sleep disturbances and mental health status.

**Methods:** An online survey was accessed by 19,740 people throughout China from February 14 to 21, 2020. The survey included the Depression Anxiety Stress Scale-21 (DASS-21) to measure psychological distress and two questions about sleep disturbances. Logistic regression analyses and moderation analysis were performed.

**Results:** (1) Among the 14,505 respondents included in analyses, 3,783 (26.08%) reported sleep disturbances at least 3 days during the past week. (2) Sleep disturbances increased the risk of depression, anxiety, and stress (*p* < 0.05). (3) Gender, age, education, occupation, frequency of attending to epidemic information, nervousness about supplies, receiving provisions of living necessities from the service department during the outbreak, number of correct responses to questions about the epidemic, and isolation/quarantine affected the risk of mental health problems among participants experiencing sleep disturbances (*p* < 0.05). (4) A moderation analysis found that sleep problems were more likely to affect depression, anxiety, and stress scores in men than women during the COVID-19 outbreak.

**Conclusion:** During the COVID-19 outbreak, 26.08% people surveyed experienced sleep disturbances, and the presence of sleep disturbances was positively related to depression, anxiety, and stress, especially among front-line anti-epidemic workers, younger people, people living in isolation/quarantine, people with a college or greater education, and males.

## Introduction

In November 2019, a novel coronavirus disease (COVID-19) was reported in Wuhan, Hubei province, China and spread quickly throughout the country (Chan et al., 2020). As of 24:00, March 1, 2020, a total of 80,026 COVID-19 cases had been confirmed and 2,803 people had died due to COVID-19-related illnesses in China (National Health Commission of the People's Republic of China, 2020). Infectious disease outbreak stress itself has negative mental health impacts (Lai et al., 2020; Liu et al., 2020), in addition to the physical harm caused to infected patients by contracting COVID-19. The negative mental health impacts may be due to COVID-19 outbreaks acting as a stressor due to fear of infection (especially in hard-hit areas), social isolation, nervousness about supply security, and uncertainty about the disease. In a study of 56,932 subjects during the COVID-19 pandemic, it was reported that 27.9% of respondents experienced depression symptoms, 31.6% experienced anxiety symptoms, 24.4% experienced acute stress symptoms, and 29.2% reported insomnia (Shi et al., 2020).

Sleep disturbances have consistently been associated with severe social crisis events. Three years after the Ya'an earthquake, 32.5% of 6,132 respondents reported difficulty falling asleep, 25.3% reported experiencing bad sleep quality (Tang et al., 2018). Furthermore, 20% of patients who recovered from the SARS infection reported negative psychological effects, including insomnia (Tsang et al., 2004), a rate higher than the insomnia rate in the Chinese population during times of non-disaster (15%) (Cao et al., 2017). Sleep disturbances have also been reported among health workers and the general public in small sample and limited population studies during the COVID-19 outbreak. For example, Zhang et al. (2020) found that 38.4% of the surveyed health workers (*N* = 927) reported insomnia symptoms compared to 30.5% of non-health workers (*N* = 1,255). At present, there are no large sample, representative population studies on sleep disturbances among the general population during the COVID-19 outbreak.

Good quality sleep is vital to both physical and mental health (Fernandez-Mendoza and Vgontzas, 2013). Sleep disturbances are associated with mental health disorders and pose a risk for depression, anxiety, and post-traumatic stress disorder (PTSD) (Alvaro et al., 2013; Del Rio João et al., 2018). Children with persistent sleep disturbances are 16 times more likely than those without sleep problems to develop psychosocial symptoms in a subclinical/clinical population (Simola et al., 2014). A longitudinal study among the general public reported a close relationship between sleep disturbances and poor distress tolerance (Kechter and Leventhal, 2019). In addition, Buysse et al. (2008) found that having a baseline sleep disturbance was a strong predictor for mental health impairment. Collectively, the above studies indicate that sleep disturbances are closely related to mental health issues in both patients and healthy individuals. Since the start of the COVID-19 outbreak, individual studies (Ahmed et al., 2020; Lai et al., 2020) and a systematic review (Vindegaard and Benros, 2020) have focused mostly on psychological distress. However, only one small sample study (Liu et al., 2020) has explored the relationship between mental health and sleep quality during the COVID-19 outbreak, finding a close relationship between sleep disturbances and post-traumatic stress symptoms (PTSS). In addition, the following have been identified as risk factors for the experience of psychological distress during the outbreak: younger age, more time spent focusing on the COVID-19 epidemic, being female, living in a hard-hit area, and being a frontline health care worker (Huang and Zhao, 2020; Lai et al., 2020; Liu et al., 2020; Zhang et al., 2020). Whether these risk factors play a role in with the relationship between sleep disturbances and mental health is therefore worth exploring. Early psychological responses to stressful events have been shown to be closely related to negative mental health outcomes in later in life, including depression and anxiety (Garfin et al., 2018). Therefore, recognition of psychological reactions to traumatic events early in life and the administration of timely and effective interventions are vital to mental health later in life. Based on the aforementioned findings, we hypothesized that sleep disturbances would be closely related to mental health issues during the COVID-19 pandemic and may be a predictive marker for mental health risk during the early, most uncertain period of the pandemic.

Prior studies have found that gender plays a significant role in the experience of sleep disturbances. For example, a review by Suh et al. (2018) found that studies consistently report a higher rate of insomnia among females than males. Females are twice as likely to suffer from sleep disruptions as males (Mong et al., 2011). During a previous earthquake crisis, sleep disturbances were more prevalent among females than males (Nakamura et al., 2020). In addition, gender differences have been reported in the experience of mental health issues. Matud and García (2019) found the incidence of mental health issues to be higher in women than men. Furthermore, sleep deprivation causes a higher level of anxiety in women than in men (Goldstein-Piekarski et al., 2018). Females have reported greater psychological distress than males during the COVID-19 pandemic (Liu et al., 2020). However, whether gender affects the relationship between sleep disturbances and mental health during the COVID-19 outbreak is not currently known and is worthy of further study. Thus, we hypothesized that there would be an effect of gender on the relationship between sleep disturbances and mental health.

The current study assessed the prevalence of sleep disturbances among different demographic groups of Chinese people during the COVID-19 pandemic. In addition, the relationship between sleep disturbances and mental health status and its' related factors were explored. The results of the current study will provide valuable information to medical staff and the government about who would best benefit from interventions directed at preventing sleep disturbances and the promotion of mental health during the COVID-19 crisis.

## Methods and Materials

### Participants and Procedure

The study survey was distributed using the Wenjuanxing platform (Chinese Online Survey Platform) via WeChat from February 14 to 21, 2020. In total, 19,740 Chinese people throughout China, including 31 provinces and autonomous regions, completed the survey. However, 5,235 participants were excluded from analysis because they were under 18 years old (N = 496) or failed the “trick” questions. As an example of a “trick” question, participants were required to list their gender in two places on the questionnaire. If the answers differed, the questionnaire was considered invalid (*N* = 4,739). In total, 14,505 participants were included in the study (age: 33.04 ± 10.10 years old; range 18–81 years). There were more female (*N* = 9,016, 62.16%) participants than male (N = 5,489, 37.84%). Most participants (*N* = 9,604, 66.21%) had a college degree or higher, while 3,195 (22.03%) had a high school degree, and 1,706 (11.76%) had a middle school education or below. Of all participants, 812 (5.60%) were front-line anti-epidemic workers, 2,617 (18.04%) were students, and 11,076 (76.36%) had other occupations. Data on sleep disturbances (difficulty falling asleep and waking early) and mental health status (depression, anxiety, and stress symptoms) were collected. The questionnaire survey was conducted anonymously to protect privacy. Informed consent was obtained from participants prior to completing the survey. All participants were informed of their right to refuse to participate in the study and their right to withdraw from the survey at any time after participating. The present study was approved by the Ethics Committee of Shenzhen Kangning Hospital (No. 2020-K001-01).

### Measurements

#### Demographic Information

Demographic data, including age, gender, education, and occupation were collected.

#### Mental Health Status (Depression, Anxiety, and Stress) Assessment

Depression, anxiety, and stress were measured by the short version Depression Anxiety Stress Scale (DASS-21) (Henry and Crawford, 2005), which was adapted from the Depression Anxiety Stress Scale (Lovibond and Lovibond, 1995). The Chinese version of the DASS-21 was revised by Gong et al. (2010). The DASS-21 is a self-reported questionnaire including three subscales with 7 items each to evaluate depression, anxiety, and stress. Each item assesses the severity of depression, anxiety, or stress experienced in the past week and is answered on a 4-point scale, from 0 (“does not apply to me at all”) to 3 (“applies to me very much or most of the time”). The total score for each symptom is categorized into five levels of severity: normal, mild, moderate, severe, and extremely severe. In the current study, depression, anxiety, and stress were analyzed as binary variables in the rate analysis and logistic regression model with a cut-off of severe and above severe (depression score: ≥21; anxiety score: ≥15; and stress score: ≥26) (Lovibond and Lovibond, 2002). In the moderation model, depression, anxiety, and stress were treated as continuous variables. The Cronbach's α was 0.914 for depression, 0.919 for the stress, and 0.917 for the anxiety in the present study, indicating a good internal consistency for each subscale of the DASS-21 in the present study.

#### Sleep Status Assessment

According to Chen et al. insomnia was defined as difficulty falling asleep or waking up early ≥3 days/week (Chen et al., 2005). Therefore, sleep disturbances were evaluated with two questions in the current study. Difficulty falling asleep was assessed by the question, “Have you had any difficulty falling asleep in the last week?” and Waking up early was assessed by the question, “Have you woken up too early or during the night in the last week?” Response options for each item included “every day,” “5–6 days/week,” “3–4 days/week,” “1–2 days/week” and “not at all.” A response of ≥3 days/week in difficulty falling asleep or waking up early was used as a cut-off for sleep disturbances.

#### Knowledge About COVID-19

An understanding of COVID-19 was assessed by the four following items. 1. COVID-19 epidemiological history: “How many days prior to the onset of illness has the average patient traveled or lived in Wuhan and surrounding areas, or other communities with case reports?” Response options included, “21 days,” “14 days” (correct choice), and “7 days” (Lauer et al., 2020); 2. Sources of infection: “What are the major sources of infection so far?” Response options included, “patients with novel coronavirus infection symptoms,” “patients with novel coronavirus infection symptoms and asymptomatic infection” (correct choice) and “patients with asymptomatic infection” (General Office of Health Commission Office of the State Administration of Traditional People's Republic of China, 2020); 3. “Who is susceptible to novel coronavirus?” Response options included, “the population is generally susceptible” (correct choice), “only the elderly and people with underlying diseases,” “infants are not susceptible,” and “healthy, middle-aged people are not susceptible” (Tian et al., 2020); 4. “As far as you know, correctly wearing a mask, washing hands frequently, and reducing the frequency of visiting crowded and enclosed spaces can reduce the risk of infection.” Response options included, “Yes” (correct choice) and “No” (Lotfi et al., 2020).

### Statistical Analyses

Data analysis was performed using SPSS 21.0 (IBM, Armonk, NY) software and Process 3.2 plug-in. The prevalence of self-reported sleep disturbances and the prevalence of depression, anxiety, and stress under different sleep disturbance severities are presented as rates (%) with 95% confidence interval (CI). Logistic regression analyses were used to explore the association between sleep disturbances and mental health status and the influencing factors on mental health status among those with sleep disturbances. A moderation analysis was performed to explore the effect of gender on sleep problems and mental health status. Statistical significance was set at *p* < 0.05.

## Results

### Prevalence of Sleep Disturbances and Demographic Distribution of Participants

Among 14,505 participants, 3,783(26.08%) reported sleep disturbances at least 3 days during the past week, 2,790 (19.23%) reported difficulty falling asleep at least 3 days during the past week and 2,973 (20.50%) reported waking up early or during the night at least 3 days during the past week. Among 5,489 male participants, 1,146 (20.88%) reported difficulty falling asleep at least 3 days during the past week and 1,203 (21.92%) reported waking early or during the night at least 3 days during the past week. Among 9,016 female participants, 1,644 (18.23%) reported difficulty falling asleep at least 3 days during the past week and 1,770 (19.63%) reported waking early or during the night at least 3 days during the past week (See Table 1).

**Table 1:** Demographic distribution by prevalence of sleep disturbances at least 3 days during the past week (*N* = 14,505).

**Characteristic**	***N***	**Difficulty falling asleep** ***N* (%, 95%CI)**	**Waking early** ***N* (%, 95%CI)**
Total	14,505 (100%)	2,790 (19.23%, 18.60–19.88%)	2,973 (20.50%, 19.85–21.16%)
**Gender**			
Male	5,489 (37.84%)	1,146 (20.88%, 19.82–21.97%)	1,203 (21.92%, 20.84–23.03%)
Female	9,016 (62.16%)	1,644 (18.23%, 17.45–19.04%)	1,770 (19.63%, 18.82–20.46%)
**Age*****, y***			
18–44	12,412 (85.57%)	2,453 (19.76%, 19.07–20.47%)	2,499 (20.13%, 19.44–20.85%)
≥45	2,093 (14.43%)	337 (16.10%,14.59–17.74%)	474 (22.65%, 20.90–24.49%)
**Education**			
Middle school or less	1,706 (11.76%)	324 (18.99%,17.20–20.92%)	399 (23.39%, 21.44–25.46%)
High school	3,195 (22.03%)	625 (19.56%,18.22–20.97%)	672 (21.03%, 19.65–22.48%)
College and above	9,604 (66.21%)	1,841 (19.17%, 18.39–19.97%)	1,902 (19.80%, 19.02–20.61%)
**Occupation**			
Front-line anti-epidemic worker	812 (5.60%)	252 (31.03%,27.95–34.30%)	274 (33.74%, 30.57–37.07%)
Student	2,617 (18.04%)	400 (15.28%,13.96–16.71%)	316 (12.07%, 10.88–13.38%)
Others	11,076 (76.36%)	2,138 (19.30%, 18.58–20.05%)	2,383 (21.51%, 20.76–22.29%)

*CI, confidence interval*.

### The Relationship Between Depression, Anxiety, and Stress and Level of Sleep Disturbances and Unadjusted Logistic Regression Analysis

The rates of severe and above severe depression, anxiety, and stress symptoms increased with the number of days/weeks of self-reported sleep disturbances. The highest rates of severe and above severe depression, anxiety, and stress symptoms were found among those who reported sleep disturbances 5–6 days during the past week (See Supplementary Table 1).

The unadjusted logistic regression analysis for predicting mental health status yielding three notable findings. (1) There was an increased risk of severe and above severe depression symptoms among participants who reported difficulty falling asleep “1–2,” “3–4,” “5–6,” and “7” days/past week compared with participants who did not report difficulty falling asleep. In addition, there was an increased risk of severe and above severe depression symptoms among participants who reported waking early “1–2,” “3–4,” “5–6,” and ‘7’ days/past week compared with who did not report waking early (See Supplementary Table 1A). (2) There was an increased risk of severe and above severe anxiety symptoms among participants who reported difficulty falling asleep “1–2,” “3–4,” “5–6,” and “7” days/past week compared with participants who did not report difficulty falling asleep. In addition, there was an increased risk of severe and above severe anxiety symptoms among participants who reported waking early “1–2,” “3–4,” “5–6,” and “7” days/past week compared with participants who did not report waking early (See Supplementary Table 1B). (3) There was an increased risk of severe and above severe stress symptoms among participants who reported difficulty falling asleep “1–2,” “3–4,” “5–6,” and “7” days/past week compared with participants who did not report difficulty falling asleep. Severe and above serve stress symptoms was also increased among participants who reported waking early “1–2,” “3–4,” “5–6,” and ‘7’ days/past week compared with participants who did not report waking early (See Supplementary Table 1C).

### Risk Factors for Mental Health Status Among Participants Experiencing Sleep Disturbances

A logistic regression analysis showed that among participants who reported sleep disturbances ≥3 days/week, gender (males > females), age (18–44 years > 45 years or older), education (college and above > middle school and below), occupation (front-line epidemic workers > students and other occupations), frequency of attending to epidemic information (≤2 and 3–6 times/d > ≥7 times/d), nervousness about supplies (Yes > No), provision of living necessities from the service department during the outbreak (Yes > No), correctly answered items on epidemic questions (0–2 items > 4 items), and living in isolation/quarantine (Yes > No) affected the risk of severe and above severe mental health problems (see Table 2 and Supplementary Table 2).

**Table 2:** Risk factors for severe and above severe depression, anxiety, and stress symptoms among participants experiencing difficulty falling asleep ≥3 days/week (*N* = 2,790).

**Factor**	**Category**	**Adjusted logistic regression for severe and above severe depression symptoms**					
		***B***	**SE**	**Wals**	**df**	***p***	**Adjusted odd ratio (95%CI)**
**Risk factors for severe and above severe depression symptoms among participants experiencing difficulty falling asleep ≥3 days/week (*****N*** **= 2,790)**							
Gender	Male						Reference
	Female	−0.52	0.09	33.343	1	<0.001	0.595 (0.499–0.709)
Age, y	18–44						Reference
	≥45	−0.508	0.162	9.834	1	0.002	0.602 (0.438–0.827)
Education	Middle school and below						Reference
	High school	−0.15	0.167	0.809	1	0.368	0.86 (0.62–1.194)
	College and above	−0.044	0.153	0.083	1	0.774	0.957 (0.71–1.291)
Occupation	Front-line anti-epidemic worker						Reference
	Students	−0.524	0.19	7.555	1	0.006	0.592 (0.408–0.861)
	Others	−0.251	0.152	2.725	1	0.099	0.778 (0.578–1.048)
Frequency of attention to epidemic information, /d	≥7						Reference
	3–6	0.525	0.102	26.39	1	<0.001	1.69 (1.383–2.065)
	≤2	0.501	0.139	13.034	1	<0.001	1.65 (1.257–2.166)
Nervousness about supplies	No						Reference
	Yes	0.839	0.243	11.956	1	0.001	2.315 (1.438–3.724)
Provision of living necessities from the service department during the outbreak	Yes						Reference
	No	−0.345	0.105	10.798	1	0.001	0.708 (0.576–0.87)
Correctly answered items about the epidemic	0–2						Reference
	3	−0.058	0.141	0.168	1	0.682	0.944 (0.715–1.245)
	4	−0.248	0.137	3.269	1	0.071	0.78 (0.596–1.021)
Living in isolation/quarantine	No						Reference
	Yes	0.621	0.091	46.873	1	<0.001	1.862 (1.558–2.224)
Likelihood ratio test: Chi-square = 195.186, df = 13, *p* < 0.001; Nagelkerke R square = 0.099							
**Factor**	**Category**	**Adjusted logistic regression for severe and above severe anxiety symptoms**					
		**B**	**SE**	**Wals**	**df**	**p**	**Adjusted odd ratios (95%CI)**
**Risk factors for severe above severe anxiety symptoms among participants experiencing difficulty falling asleep ≥3 days/week (N = 2 790)**							
Gender	Male						Reference
	Female	−0.474	0.083	32.364	1	0	0.623 (0.529–0.733)
Age, y	18–44						Reference
	≥45	−0.439	0.144	9.311	1	0.002	0.645 (0.486–0.855)
Education	Middle school and below						Reference
	High school	−0.071	0.156	0.208	1	0.649	0.931 (0.686–1.264)
	College and above	0.156	0.143	1.193	1	0.275	1.169 (0.883–1.548)
Occupation	Front-line anti-epidemic worker						Reference
	Student	−0.436	0.175	6.174	1	0.013	0.647 (0.459–0.912)
	Others	−0.245	0.144	2.902	1	0.088	0.783 (0.591–1.038)
Frequency of attention to epidemic information, /d	≥7						Reference
	3–6	0.223	0.092	5.872	1	0.015	1.25 (1.044–1.497)
	≤2	0.414	0.126	10.803	1	0.001	1.513 (1.182–1.936)
Nervousness about supplies	No						Reference
	Yes	1.063	0.222	22.843	1	0	2.894 (1.872–4.475)
Provision of living necessities from the service department during the outbreak	Yes						Reference
	No	−0.421	0.095	19.518	1	0	0.656 (0.544–0.791)
Correctly answered items about the epidemic	0–2						Reference
	3	−0.102	0.132	0.604	1	0.437	0.903 (0.697–1.169)
	4	−0.221	0.127	3.024	1	0.082	0.802 (0.625–1.028)
Living in isolation/quarantine	No						Reference
	Yes	0.631	0.084	55.76	1	0	1.879 (1.592–2.217)
Likelihood ratio test: Chi-square = 217.454, df = 13, *p* < 0.001; Nagelkerke *R* square = 0.103.							
**Risk factors for severe and above severe stress symptoms among participants experiencing difficulty falling asleep** **≥3 days/week (*****N*** **=** **2,790)**							
Gender	Male						Reference
	Female	−0.484	0.097	24.996	1	0	0.616 (0.51–0.745)
Age, y	18–44						Reference
	≥45	−0.37	0.175	4.479	1	0.034	0.691 (0.49–0.973)
Education	Middle school and below						Reference
	High school	−0.003	0.187	0	1	0.988	0.997 (0.691–1.44)
	College and above	0.112	0.171	0.425	1	0.514	1.118 (0.799–1.565)
Occupation	Front-line anti-epidemic workers						Reference
	Students	−0.741	0.201	13.567	1	0	0.476 (0.321–0.707)
	Others	−0.445	0.155	8.278	1	0.004	0.641 (0.473–0.868)
Frequency of attention to epidemic information, /d	≥7						Reference
	3–6	0.577	0.111	27.097	1	0	1.78 (1.433–2.212)
	≤ 2	0.427	0.153	7.738	1	0.005	1.532 (1.134–2.069)
Nervousness about supplies	No						Reference
	Yes	1.037	0.297	12.157	1	0	2.821 (1.575–5.054)
Provision of living necessities from the service department during the outbreak	Yes						Reference
	No	−0.475	0.118	16.31	1	0	0.622 (0.494–0.783)
Correctly answered items about the epidemic	0–2						Reference
	3	0.123	0.161	0.585	1	0.444	1.131 (0.825–1.549)
	4	0.093	0.155	0.36	1	0.548	1.097 (0.81–1.487)
Living in isolation/quarantine	No						Reference
	Yes	0.582	0.097	35.718	1	0	1.79 (1.479–2.167)
Likelihood ratio test: Chi-square = 187.025, df = 13, *p* < 0.001; Nagelkerke R square = 0.101							

*Wals, Wald statistic; CI, confidence interval*.

### Gender as a Moderator in the Relationship Between Sleep Disturbances and Mental Health

A moderation analysis to test the influence of gender on the relationship between sleep problems and depression, anxiety, and stress symptoms was conducted adjusting for age, education, occupation, frequency of attention to epidemic information, nervousness about supplies, provision of living necessities from the service department during the outbreak, correctly answered items on epidemic questions, and living in isolation/quarantine. The results showed that gender moderated the relationship between both types of sleep disturbances and depression, anxiety, and stress symptom scores during the outbreak (all *p* < 0.05). Males with sleep disturbances were more likely to report higher levels of depression, anxiety, and stress symptoms.

## Discussion

The COVID-19 pandemic is a serious public health concern that has spread worldwide, causing psychological distress and symptoms of mental illnesses (Ahmed et al., 2020; Bao et al., 2020; Huang and Zhao, 2020; Lai et al., 2020; Rajkumar, 2020). The findings from the current study indicate that during the period of February 14–21, 2020, 26.08% of respondents from the general public in China reported sleep disturbances at least 3 days during the past week, a higher rate than during non-disaster times (15.0%) based on the latest meta-analysis data from China (Cao et al., 2017). The findings in our study are consistent with previous studies (Tsang et al., 2004; Tang et al., 2018) and recent studies (Huang and Zhao, 2020; Shi et al., 2020; Voitsidis et al., 2020), indicating that the prevalence of sleep problems is high during health epidemics and other traumatic events. However, it may be worth noting that prior studies have used different assessment tools to measure the prevalence rate of sleep problems, and therefore, a direct comparison of sleep disorders between the current study and other studies may not be valid.

The current study found an increased risk of severe and above severe depression, anxiety, and stress in participants who reported sleep disturbances compared with participants who reported no sleep disturbances. Sleep quality can affect the immune system, the endocrine system, and neurobiological functions (Lange et al., 2010; Baglioni et al., 2016), and is therefore an important dimension for both physical and mental health. In addition, the neural pathways associated with sleep are closely related to, and in part overlap with, neural pathways of affect, cognition and other important brain functions (Baglioni et al., 2016). The bidirectional relationship between sleep disturbances and mental health issues (such as anxiety and depression) has been previously well-studied (Alvaro et al., 2013; Del Rio João et al., 2018). Stress and negative emotions can adversely affect sleep (Dahlgren et al., 2005), and conversely, disturbed sleep can exacerbate the occurrence and development of mental health problems (Lin et al., 2018; Xiao et al., 2020). Moreover, poor sleep quality is independently and potentially etiologically, associated with anxiety (Johnson et al., 2006). A recent study (Rajkumar, 2020) reported high levels of anxiety and depression (16–28%) and stress (8%) during the COVID-19 outbreak, and that these mental health problems may be associated with sleep disturbances. The results of the present study highlight the association between sleep and mental health issues and indicate the necessity of rapid identification and intervention focusing on sleep issues to reduce psychological problems during public health events. Prior research (Xiao et al., 2020) found a positive effect of social support on sleep quality during the COVID-19 outbreak. Other interventions that benefit sleep, such as relaxation, daytime light exposure, physical activity, and reducing bedtime mobile phone use are also recommended (Tamrat et al., 2014; Exelmans and Van den Bulck, 2016; Baldursdottir et al., 2017).

After logistic regression analysis, we found a number of risk factors associated with mental health status among those reporting sleep disturbances. Consistent with previous research during epidemics (Huang and Zhao, 2020; Liang et al., 2020; Zhang et al., 2020), young people (18–44 years) and front-line epidemic workers were at greater risk for severe and above severe mental health problems. Furthermore, participants with a higher education level were more anxious and depressed, likely because they live in more prosperous cities or areas where the epidemic is spread more easily due to high population density, and because they think more about the epidemic situation. People who reported anxiety about supplies, and who were living in isolation/quarantine were also more likely to develop mental health issues. Those living in isolation/quarantine worry about their epidemiological contact history and experience loneliness and boredom due to a lack of communication with others (Hawryluck et al., 2004). In addition, those living in isolation would be especially impacted by anxiety over insufficient supplies. Those receiving provisions from the service department were also at a high risk for mental health issues, and this may be related to living in isolation/quarantine. Those infected with COVID-19 or living in high-risk epidemic areas are isolated or quarantined by necessity and receive their basic supplies from the community. These findings underline the importance of psychological intervention, especially for vulnerable populations, including front-line epidemic workers, younger people, and those who are isolated or in quarantine. Moreover, these results suggest that the government should take timely measures to ensure basic living needs are met and to increase social support, such as the provision of a mental assistance hotline. In contrast to another study conducted during the COVID-19 pandemic (Huang and Zhao, 2020), we found that people who paid less attention to epidemic information were more likely to develop mental health problems. These results may be due to fear and anxiety of the unknown. In support, we also found that people with less knowledge of the epidemic (scoring lower on questions related to the epidemic) were also at greater risk for mental health problems. These findings suggest the value of internet services and social media to spread true and useful information and to avoid potentially harmful rumors.

Though we found that sleep problems affected mental health status in both males and females, we found that males with sleep disturbances were more vulnerable to mental health issues than females during the early outbreak of COVID-19 in China, suggesting that the relationship between sleep disturbances and mental health status is stronger in males. Males and females respond differently to stress, and this sex difference likely leads to the higher susceptibility to stress-related mental illness reported in females compared to males (Bangasser and Wicks, 2017). Contradictory to our results, prior studies examining gender differences in mental health issues found that females scored higher in emotional coping, psychological distress, and chronic stress than males, and more vulnerable to mental health issues when lost sleep (Goldstein-Piekarski et al., 2018; Matud and García, 2019). One possible explanation for the apparent discrepancy is that females may be more vulnerable to chronic mild stress in daily life while males may be more affected by acute stress, such as that brought on by COVID-19 (Dalla et al., 2005). Females are more likely than males to cope by confiding and asking for help (Lim Vivien and Teo Thompson, 1996) and this coping style may be protective against the effects of pandemic-associated stress. Higher psychological resilience has been found to be associated with a better positive coping style (Wu et al., 2020). Nevertheless, the current findings underscore the importance of attending to mental health in males, especially during times of acute stress. These findings also point to a particular need to improve psychological resilience and positive coping skills in males. Further research is needed to better understand coping styles typical of males.

## Limitations

The current study had limitations that must be considered. First, because the research was conducted with a convenience sampling method through online questionnaires on the WeChat platform, a sample bias and response bias favoring individuals with a higher education, who are more likely to use WeChat more frequently, may exist. Second, this is a cross-sectional survey that was conducted at the early stage of the outbreak of COVID-19 in China. The data capture mental health status specifically during that time. Longitudinal follow-up studies are recommended to examine the dynamic processes of people's mental health status throughout the outbreak. Thirdly, we investigated sleep disturbances during the week prior to participation in the study which may have yielded a higher rate of sleep disturbances than studies that defined sleep disturbances as symptoms that last for 1 month or longer. Lastly, specialized sleep assessment scales were not used, leading to the exclusion of details, such as the severity of sleep issues, limiting our understanding of the experienced sleep disturbances.

## Conclusion

The current study identified a high rate of sleep disturbances in the Chinese population during the COVID-19 outbreak and that the presence of sleep disturbances increased the risk of mental health problems, especially in front-line epidemic workers, people who were quarantined or isolated, young people, those with higher education, and males. The presented findings highlight the importance of intervention directed at those experiencing sleep issues to reduce mental health problems during a public health crisis. In particular, vulnerable populations should be closely monitored. The current findings provide a reference for the development of mental health intervention policies during epidemic/pandemic events.

## Data Availability Statement

The raw data supporting the conclusions of this article will be made available by the authors, without undue reservation.

## Ethics Statement

The studies involving human participants were reviewed and approved by the ethical committee of Shenzhen Kangning Hospital. Written informed consent for participation was not required for this study in accordance with the national legislation and the institutional requirements.

## Author Contributions

XC, YH, and JL conceived and designed framework of this study. XC, YH, and JG collected data. JL executed the statistical analysis. XC and YH drafted the manuscript. XL and JL revised the manuscript. All authors read and approved the final manuscript.

## Conflict of Interest

The authors declare that the research was conducted in the absence of any commercial or financial relationships that could be construed as a potential conflict of interest.
