# Supplementary material for: Epidemiology of Sleep Disturbances and Their Effect on Psychological Distress During the COVID-19 Outbreak: A Large National Study in China
Source: Front Psychol. 2021 May 28;12:615867. doi: 10.3389/fpsyg.2021.615867 (PMC8220290; doi:10.3389/fpsyg.2021.615867)
Supplement: Supplementary file 1 [file Table_1.docx]

**Supplemental Table 1**. The severe and above severe depression, anxiety, and stress symptom rates by level of sleep disturbances and unadjusted logistic regression results for predicting mental health status (N=14,505).

**Supplemental Table 1A**. The severe and above severe depression symptom rates by level of sleep disturbances and unadjusted logistic regression results for predicting severe and above severe depression symptoms (N=14,505).

| Independent variable | Days/ past week | N, n (%,95%CI) | Unadjusted logistic regression for predicting severe and above severe depression symptom | | | | | |
| --- | --- | --- | --- | --- | --- | --- | --- | --- |
|  |  |  | B | SE | Wals | *df* | *p* | odds ratio (95% confident intervals) |
| Difficulty falling asleep | 0 | 7859,278 (3.54,3.15-3.97) | Reference | | | | | |
|  | 1-2 | 3856,308 (7.99,7.17-8.89) | 0.862 | 0.085 | 102.319 | 1 | <0.001 | 2.367 (2.003-2.797) |
|  | 3-4 | 1110,196 (17.66,15.53-20.01) | 1.766 | 0.1 | 314.25 | 1 | <0.001 | 5.848 (4.811-7.109) |
|  | 5-6 | 579,205 (35.41,31.62-39.39) | 2.705 | 0.106 | 648.398 | 1 | <0.001 | 14.947 (12.138-18.407) |
|  | 7 | 1101,333 (30.25,27.60-33.02) | 2.47 | 0.09 | 759.455 | 1 | <0.001 | 11.824 (9.919-14.095) |
|  | Likelihood ratio test: Chi-square=1155.003, *df*=4, *p*<0.001; Nagelkerke R square=0.168 | | | | | | | |
| Waking early | 0 | 6971,256 (3.67,3.26-4.14) | Reference | | | | | |
|  | 1-2 | 4561,342 (7.50,6.77-8.30) | 0.754 | 0.085 | 78.863 | 1 | <0.001 | 2.126 (1.800-2.511) |
|  | 3-4 | 1180,229 (19.41,17.25-21.76) | 1.843 | 0.097 | 358.592 | 1 | <0.001 | 6.316 (5.219-7.644) |
|  | 5-6 | 508,179 (35.24,31.21-39.49) | 2.658 | 0.113 | 557.222 | 1 | <0.001 | 14.271 (11.445-17.796) |
|  | 7 | 1285,314 (24.44,22.16-26.86) | 2.138 | 0.091 | 552.737 | 1 | <0.001 | 8.482 (7.098-10.137) |
|  | Likelihood ratio test: Chi-square=970.276, *df*=4, *p*<0.001; Nagelkerke R square=0.142 | | | | | | | |

Note: Wals: Wald statistic

**Supplemental Table 1B**. The severe and above severe anxiety symptom rates by level of sleep disturbances and unadjusted logistic regression results for predicting severe and above severe anxiety symptoms (N=14,505).

| Independent variable | Days/ past week | N, n (%,95%CI) | Unadjusted logistic regression for predicting severe and above severe anxiety symptom | | | | | |
| --- | --- | --- | --- | --- | --- | --- | --- | --- |
|  |  |  | B | SE | Wals | *df* | *p* | odds ratio (95% confident intervals) |
| Difficulty falling asleep | 0 | 7859,400 (5.09,4.63-5.60) | Reference | | | | | |
|  | 1-2 | 3856,510 (13.23,12.19-14.33) | 1.045 | 0.07 | 222.978 | 1 | <0.001 | 2.842 (2.478-3.260) |
|  | 3-4 | 1110,296 (26.67,24.15-29.35) | 1.914 | 0.085 | 505.987 | 1 | <0.001 | 6.781 (5.739-8.012) |
|  | 5-6 | 579,265 (45.77,41.75-49.84) | 2.756 | 0.098 | 791.86 | 1 | <0.001 | 15.738 (12.989-19.068) |
|  | 7 | 1101,436 (39.60,36.75-42.52) | 2.504 | 0.08 | 974.569 | 1 | <0.001 | 12.226 (10.448-14.307) |
|  | Likelihood ratio test: Chi-square= 1551.277, df=4, p<0.001; Negelkerke R square=0.188 | | | | | | | |
| Waking early | 0 | 6971,381 (5.47,4.96-6.02) | Reference | | | | | |
|  | 1-2 | 4561,558 (12.23,11.31-13.22) | 0.88 | 0.069 | 160.744 | 1 | <0.001 | 2.411 (2.104-2.762) |
|  | 3-4 | 1180,329 (27.88,25.40-30.51) | 1.9 | 0.084 | 516.461 | 1 | <0.001 | 6.687 (5.676-7.878) |
|  | 5-6 | 508,234 (46.06,41.77-50.41) | 2.693 | 0.103 | 677.658 | 1 | <0.001 | 14.772 (12.061-18.091) |
|  | 7 | 1285,405 (31.52,29.04-34.11) | 2.074 | 0.08 | 674.318 | 1 | <0.001 | 7.960 (6.807-9.310) |
|  | Likelihood ratio test: Chi-square= 1245.485, df=4, p<0.001; Negelkerke R square=0.152 | | | | | | | |

Note: Wals: Wald statistic

**Supplemental Table 1C**. The severe and above severe stress symptom rates by level of sleep disturbances and unadjusted logistic regression results for predicting severe and above severe stress symptoms (N=14,505).

| Independent variable | Days/ past week | N, n (%,95%CI) | | Unadjusted logistic regression for predicting severe and above severe stress symptom | | | | | |
| --- | --- | --- | --- | --- | --- | --- | --- | --- | --- |
|  |  |  |  | B | SE | Wals | *df* | *p* | odds ratio (95% confident intervals) |
| Difficulty falling asleep | 0 | 7859,204 (2.60,2.27-2.97) | | Reference | | | | | |
|  | 1-2 | 3856,228 (5.91,5.21-6.70) | | 0.858 | 0.098 | 75.922 | 1 | <0.001 | 2.358 (1.944-2.860) |
|  | 3-4 | 1110,161 (14.50,12.56-16.70) | | 1.851 | 0.111 | 278.606 | 1 | <0.001 | 6.366 (5.122-7.912) |
|  | 5-6 | 579,152 (26.25,22.83-29.98) | | 2.592 | 0.118 | 481.525 | 1 | <0.001 | 13.358 (10.597-16.838) |
|  | 7 | 1101, 278 (25.25,22.77-27.90) | | 2.54 | 0.099 | 655.156 | 1 | <0.001 | 12.675 (10.435-15.396) |
|  | Likelihood ratio test: Chi-square=943.342, df=4, p<0.001; Negelkerke R square=0.158 | | | | | | | | |
| Waking early | 0 | | 6971,188 (2.70,2.34-3.10) | Reference | | | | | |
|  | 1-2 | | 4561,255 (5.59,4.96-6.30) | 0.759 | 0.098 | 59.918 | 1 | <0.001 | 2.137 (1.763-2.590) |
|  | 3-4 | | 1180,175 (14.83,12.92-16.97) | 1.838 | 0.11 | 277.386 | 1 | <0.001 | 6.283 (5.061-7.799) |
|  | 5-6 | | 508,141 (27.76,24.04-31.81) | 2.629 | 0.124 | 452.272 | 1 | <0.001 | 13.862 (10.879-17.662) |
|  | 7 | | 1285,264 (20.54,18.43-22.84) | 2.233 | 0.101 | 487.296 | 1 | <0.001 | 9.329 (7.651-11.375) |
|  | Likelihood ratio test: Chi-square=805.937, df=4, p<0.001; Nagelkerke R square=0.135 | | | | | | | | |

Note: Wals: Wald statistic

**Supplemental Table 2.** Risk factors for severe and above severe depression, anxiety, and stress symptoms among participants experiencing waking early ≥3 days/week (n=2,973).

**Supplemental Table 2A.** Risk factors for severe and above severe depression symptoms among participants experiencing waking early ≥3 days/week (n=2,973).

|  |  | B | SE | Wals | *df* | *p* | Adjusted logistic regression for severe and above severe depression symptoms  Adjusted odd ratios (95% confident intervals） |
| --- | --- | --- | --- | --- | --- | --- | --- |
| Gender | Male |  |  |  |  |  | Reference |
|  | Female | -0.5 | 0.091 | 30.443 | 1 | **<0.001** | 0.607(0.508-0.724) |
| Age, y | 18-44 |  |  |  |  |  | Reference |
|  | ≥45 | -0.725 | 0.156 | 21.563 | 1 | **<0.001** | 0.484(0.357-0.658) |
| Education | Middle school and below |  |  |  |  |  | Reference |
|  | High school | 0.308 | 0.173 | 3.163 | 1 | 0.075 | 1.361(0.969-1.911) |
|  | College and above | 0.32 | 0.16 | 4.014 | 1 | 0.045 | 1.378(1.007-1.885) |
| Occupation | Front-line anti-epidemic workers |  |  |  |  |  | Reference |
|  | Students | -0.445 | 0.197 | 5.072 | 1 | 0.024 | 0.641(0.435-0.944) |
|  | Others | -0.29 | 0.147 | 3.864 | 1 | 0.049 | 0.748(0.561-0.999) |
| Frequency of attention to epidemic information, /d | ≥7 |  |  |  |  |  | Reference |
|  | 3-6 | 0.34 | 0.101 | 11.269 | 1 | 0.001 | 1.404(1.152-1.712) |
|  | ≤2 | 0.499 | 0.139 | 12.885 | 1 | **<0.001** | 1.648(1.254-2.164) |
| Nervousness about supplies | No |  |  |  |  |  | Reference |
|  | Yes | 0.969 | 0.246 | 15.493 | 1 | **<0.001** | 2.635(1.626-4.268) |
| Provision of living necessities from the service department during the outbreak | Yes |  |  |  |  |  | Reference |
|  | No | -0.408 | 0.106 | 14.836 | 1 | **<0.001** | 0.665(0.54-0.818) |
| Correctly answered items about the epidemic | 0-2 |  |  |  |  |  | Reference |
|  | 3 | -0.248 | 0.14 | 3.14 | 1 | 0.076 | 0.78(0.593-1.027) |
|  | 4 | -0.412 | 0.137 | 9.045 | 1 | 0.003 | 0.662(0.506-0.866) |
| Living in isolation/quarantine | No |  |  |  |  |  | Reference |
|  | Yes | 0.768 | 0.091 | 71.65 | 1 | **<0.001** | 2.155(1.804-2.575) |
| Likelihood ratio test: Chi-square=253.889, df=13, *p*<0.001; Nagelkerke R square=0.122 | | | | | | | |

Note: Wals: Wald statistic

**Supplemental Table 2B.** Risk factors for severe and above severe anxiety symptoms among participants experiencing waking early ≥3 days/week (n=2,973).

|  |  | B | SE | Wals | *df* | *p* | Adjusted logistic regression for severe and above severe anxiety symptoms  Adjusted odd ratios (95% confident intervals） |
| --- | --- | --- | --- | --- | --- | --- | --- |
| Gender | Male |  |  |  |  |  | Reference |
|  | Female | -0.532 | 0.084 | 40.518 | 1 | **<0.001** | 0.588(0.499-0.692) |
| Age, y | 18-44 |  |  |  |  |  | Reference |
|  | ≥45 | -0.641 | 0.136 | 22.163 | 1 | **<0.001** | 0.527(0.403-0.688) |
| Education | Middle school and below |  |  |  |  |  | Reference |
|  | High school | 0.169 | 0.156 | 1.168 | 1 | 0.28 | 1.184(0.872-1.609) |
|  | College and above | 0.363 | 0.143 | 6.412 | 1 | 0.011 | 1.438(1.085-1.904) |
| Occupation | Front-line anti-epidemic workers |  |  |  |  |  | Reference |
|  | Students | -0.333 | 0.181 | 3.372 | 1 | 0.066 | 0.717(0.502-1.023) |
|  | Others | -0.356 | 0.14 | 6.498 | 1 | 0.011 | 0.7(0.533-0.921) |
| Frequency of attention to epidemic information, /d | ≥7 |  |  |  |  |  | Reference |
|  | 3-6 | 0.314 | 0.092 | 11.535 | 1 | 0.001 | 1.368(1.142-1.64) |
|  | ≤2 | 0.556 | 0.128 | 19 | 1 | **<0.001** | 1.744(1.358-2.24) |
| Nervousness about supplies | No |  |  |  |  |  | Reference |
|  | Yes | 0.955 | 0.209 | 20.937 | 1 | **<0.001** | 2.598(1.726-3.91) |
| Provision of living necessities from the service department during the outbreak | Yes |  |  |  |  |  | Reference |
|  | No | -0.426 | 0.096 | 19.874 | 1 | **<0.001** | 0.653(0.542-0.788) |
| Correctly answered items about the epidemic | 0-2 |  |  |  |  |  | Reference |
|  | 3 | -0.133 | 0.13 | 1.036 | 1 | 0.309 | 0.876(0.679-1.131) |
|  | 4 | -0.366 | 0.128 | 8.202 | 1 | 0.004 | 0.694(0.54-0.891) |
| Living in isolation/quarantine | No |  |  |  |  |  | Reference |
|  | Yes | 0.686 | 0.085 | 65.733 | 1 | **<0.001** | 1.986(1.682-2.344) |
| Likelihood ratio test: Chi-square=296.156, df=13, *p*<0.001; Nagelkerke R square=0.132 | | | | | | | |

Note: Wals: Wald statistic

**Supplemental Table 2C.** Risk factors for severe and above severe stress symptoms among participants experiencing waking early ≥3 days/week (n=2973).

|  |  | B | SE | Wals | *df* | *p* | Adjusted logistic regression for severe and above severe stress symptoms  Adjusted odd ratios (95% confident intervals） |
| --- | --- | --- | --- | --- | --- | --- | --- |
| Gender | Male |  |  |  |  |  | Reference |
|  | Female | -0.521 | 0.098 | 28.533 | 1 | **<0.001** | 0.594(0.49-0.719) |
| Age, y | 18-44 |  |  |  |  |  | Reference |
|  | ≥45 | -0.702 | 0.174 | 16.319 | 1 | **<0.001** | 0.496(0.353-0.697) |
| Education | Middle school and below |  |  |  |  |  | Reference |
|  | High school | 0.219 | 0.193 | 1.287 | 1 | 0.257 | 1.245(0.853-1.819) |
|  | College and above | 0.298 | 0.177 | 2.836 | 1 | 0.092 | 1.348(0.952-1.907) |
| Occupation | Front-line anti-epidemic workers |  |  |  |  |  | Reference |
|  | Students | -0.755 | 0.211 | 12.797 | 1 | **<0.001** | 0.47(0.311-0.711) |
|  | Others | -0.515 | 0.15 | 11.852 | 1 | 0.001 | 0.598(0.446-0.801) |
| Frequency of attention to epidemic information, /d | ≥7 |  |  |  |  |  | Reference |
|  | 3-6 | 0.489 | 0.111 | 19.482 | 1 | **<0.001** | 1.631(1.312-2.026) |
|  | ≤2 | 0.521 | 0.153 | 11.561 | 1 | 0.001 | 1.684(1.247-2.275) |
| Nervousness about supplies | No |  |  |  |  |  | Reference |
|  | Yes | 1.114 | 0.297 | 14.117 | 1 | **<0.001** | 3.047(1.704-5.448) |
| Provision of living necessities from the service department during the outbreak | Yes |  |  |  |  |  | Reference |
|  | No | -0.512 | 0.119 | 18.614 | 1 | **<0.001** | 0.599(0.475-0.756) |
| Correctly answered items about the epidemic | 0-2 |  |  |  |  |  | Reference |
|  | 3 | -0.054 | 0.159 | 0.115 | 1 | 0.734 | 0.948(0.695-1.293) |
|  | 4 | -0.062 | 0.154 | 0.164 | 1 | 0.685 | 0.94(0.695-1.271) |
| Living in isolation/quarantine | No |  |  |  |  |  | Reference |
|  | Yes | 0.596 | 0.098 | 37.05 | 1 | **<0.001** | 1.815(1.498-2.199) |
| Likelihood ratio test: Chi-square=229.399, df=13 *p*<0.001; Nagelkerke R square=0.118 | | | | | | | |

Note: Wals: Wald statistic
